# Supplementary figures and images for: Aerodynamic Characteristics of a Feathered Dinosaur Measured Using Physical Models. Effects of Form on Static Stability and Control Effectiveness
Source: PLoS One. 2014 Jan 15;9(1):e85203. doi: 10.1371/journal.pone.0085203 (PMC3893193; doi:10.1371/journal.pone.0085203)

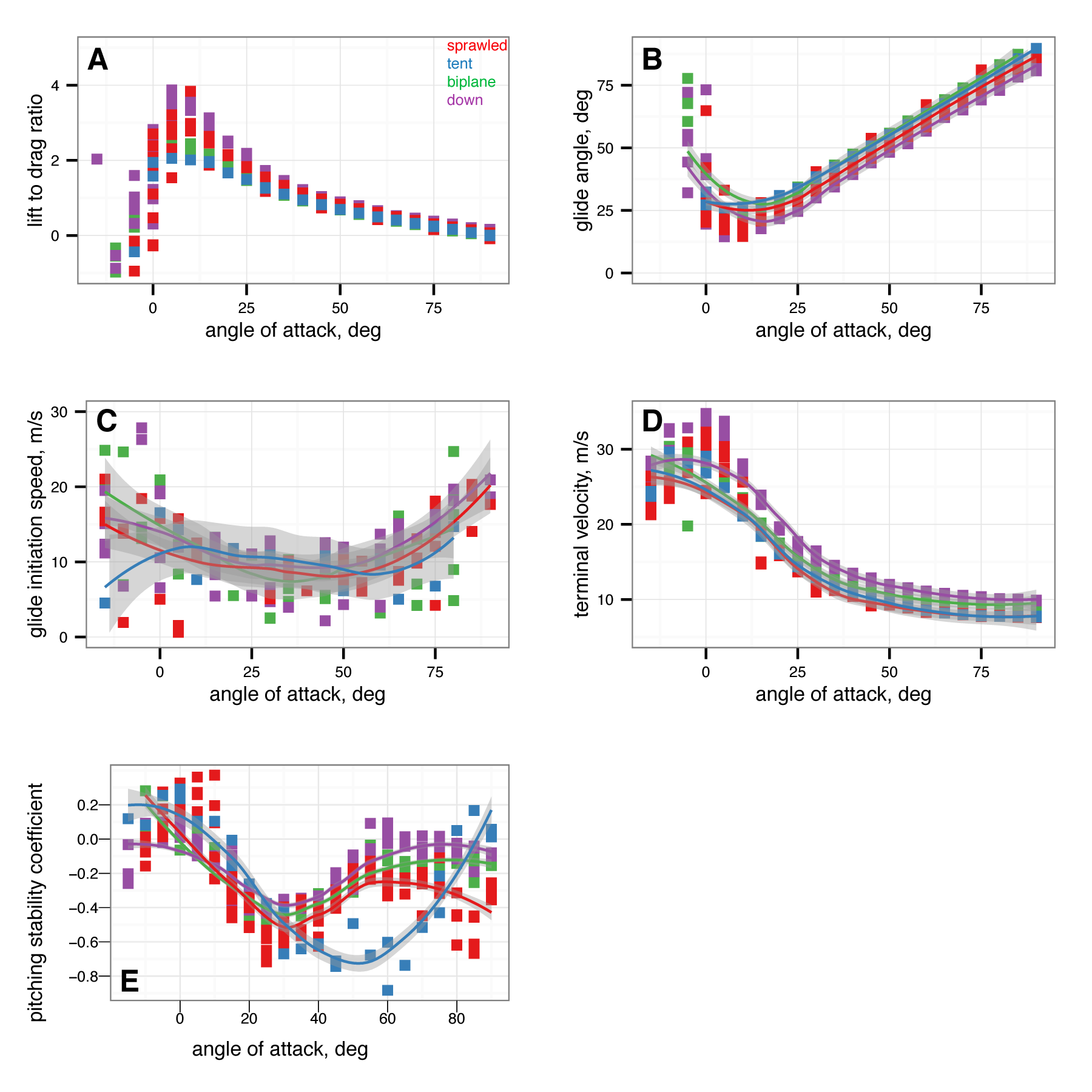

Supplement: Figure S1 — Simple gliding metrics after [65] . Red is sprawled, blue is tent, green is biplane, purple is down. from -15 to 90 in 5 increments, with five or more replicates per treatment. A, Lift to drag ratio. B, Glide angle. C, Minimum glide speed. D, Terminal velocity (at which , assuming stability). e: Pitching stability coefficient (note pitching moment must also be zero for stable equilibrium). (TIF) [file pone.0085203.s001.tif]

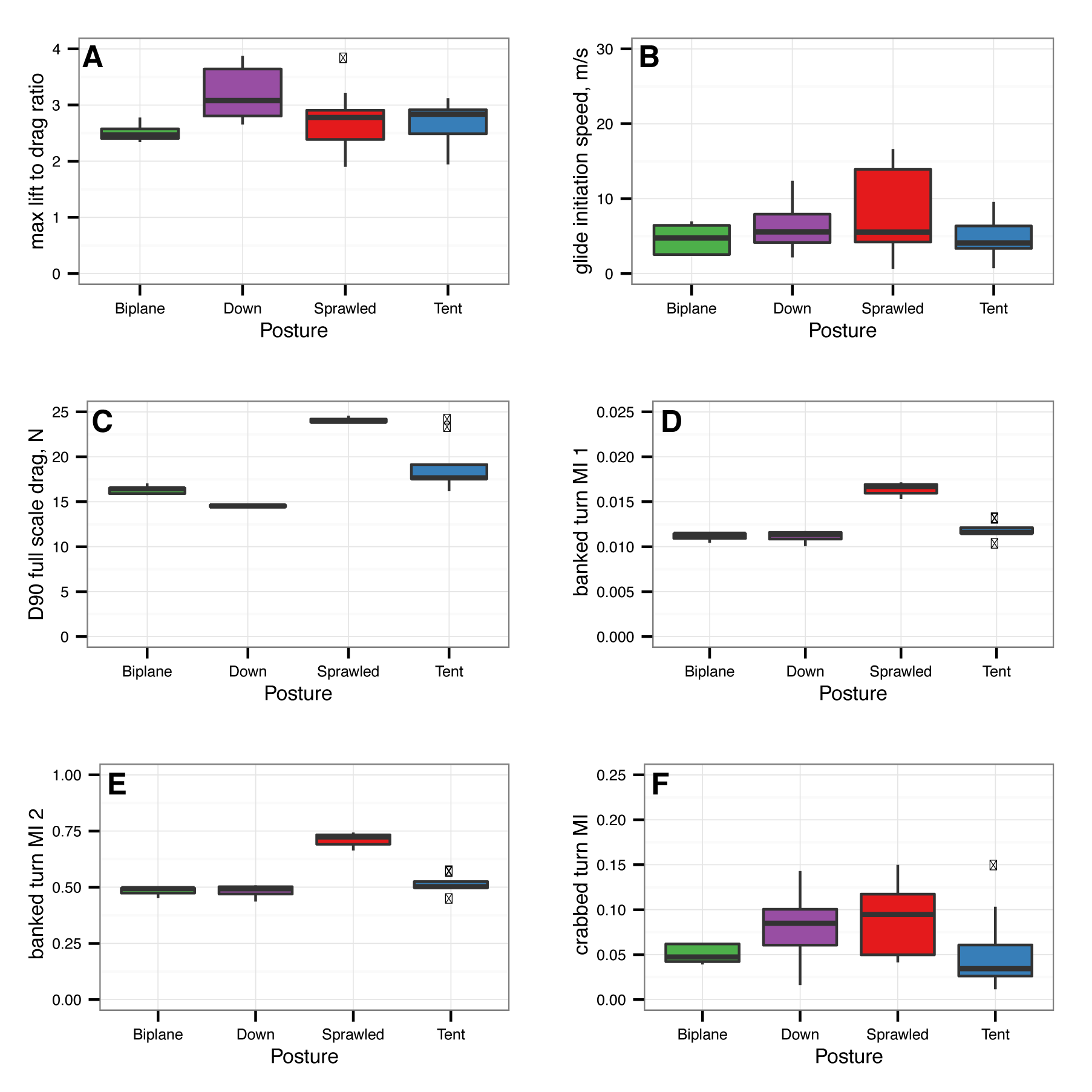

Supplement: Figure S2 — Comparison of simple glide metrics after [65] suggests the metrics are not informative. Red is sprawled, blue is tent, green is biplane, purple is down. A, Maximum lift to drag ratio, by posture, without regard to stability. [65]'s minimum ratio is never achieved because the models are not stable at the point where is maximum. There is no difference in maximum lift to drag ratio among postures (Kruskal-Wallis, ). B, Minimum glide initiation speed, by posture, without regard to stability. The minimum speed is never achieved because the models are not stable at the point where is lowest. There is no difference in among postures (Kruskal-Wallis, ). C, “Parachuting” drag, , by posture, without regard to stability. This drag is never achieved because the baseline postures are not stable at a 90 angle of attack. There are significant differences in among postures (Kruskal-Wallis, ); sprawled position has higher parachuting drag. D-E, banked turn maneuvering indices suggest sprawled posture may execute banked turns better than others, but posture is not stable. F, baseline postures not different in crabbed turn performance. (TIF) [file pone.0085203.s002.tif]

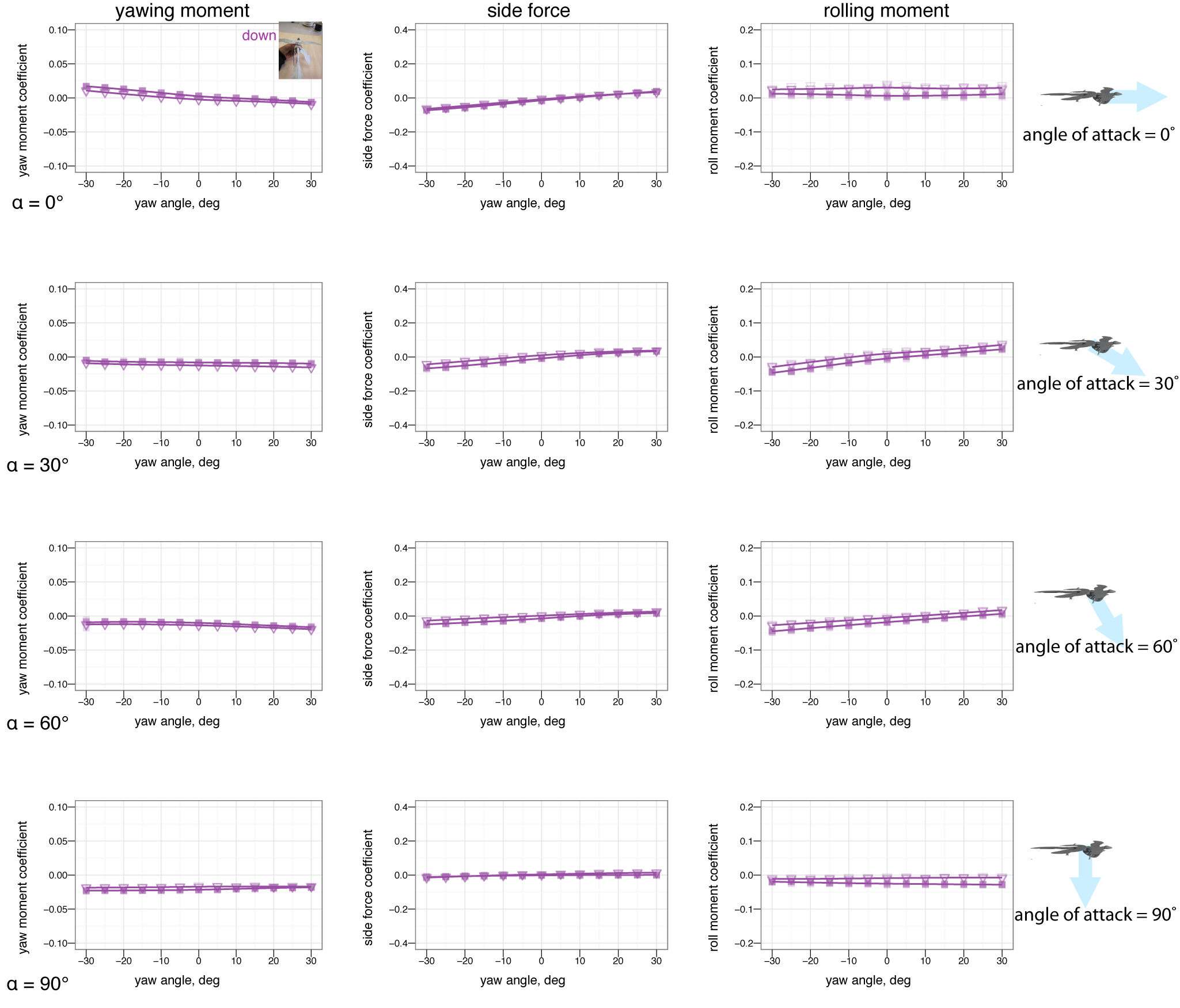

Supplement: Figure S3 — Asymmetric leg dihedral (leg dégagé , see inset) effect on yaw without leg or tail feathers. Baseline down position (solid square) versus one leg at 45 dihedral (down arrow). Without leg or tail feathers, the surfaces have little aerodynamic effect. (TIF) [file pone.0085203.s003.tif]

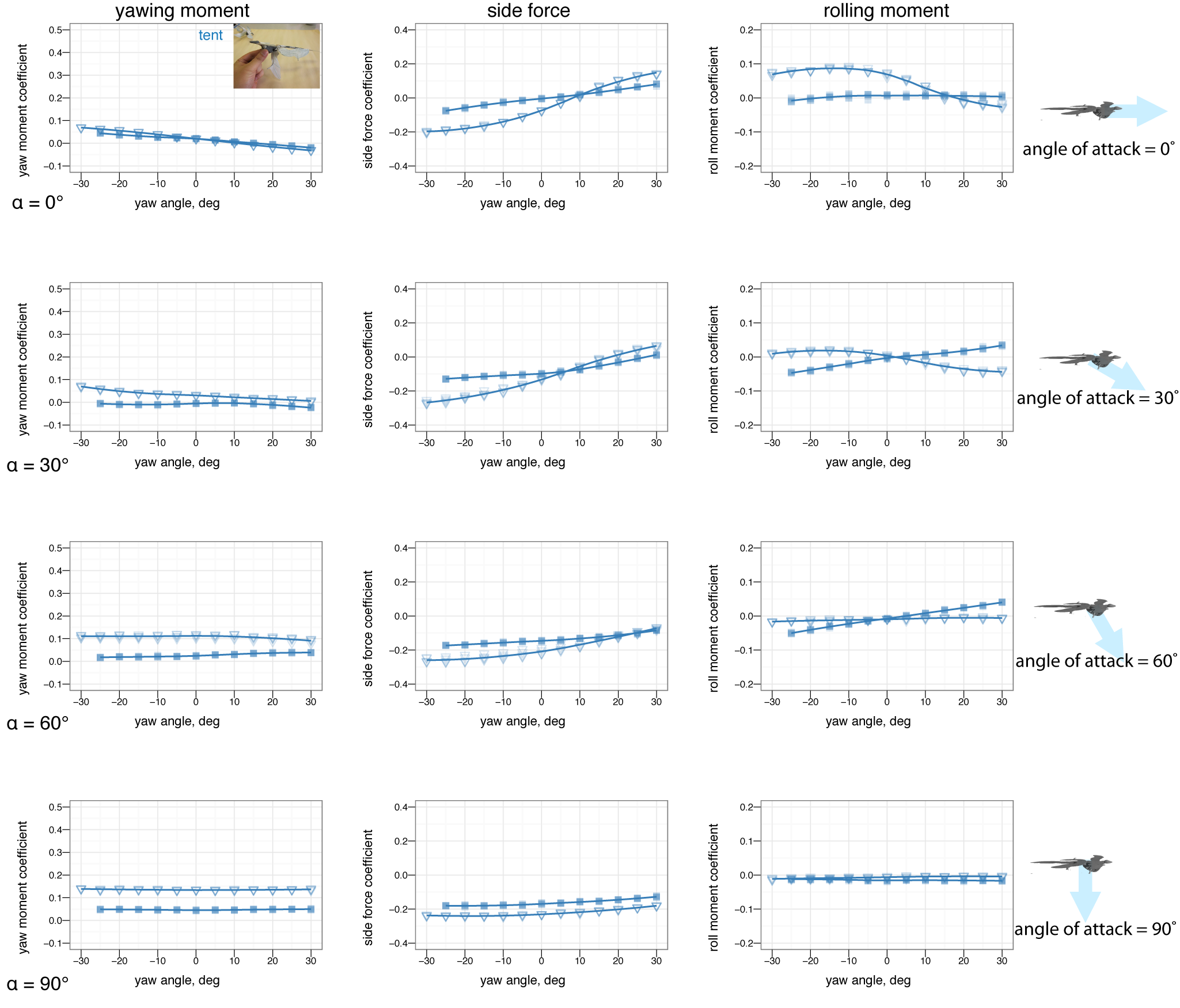

Supplement: Figure S4 — Asymmetric one leg down (leg arabesque ) effect on yaw. Baseline tent position (solid square) versus one leg at 90 mismatch (down arrow). Placing one leg down has very little effect. (TIF) [file pone.0085203.s004.tif]

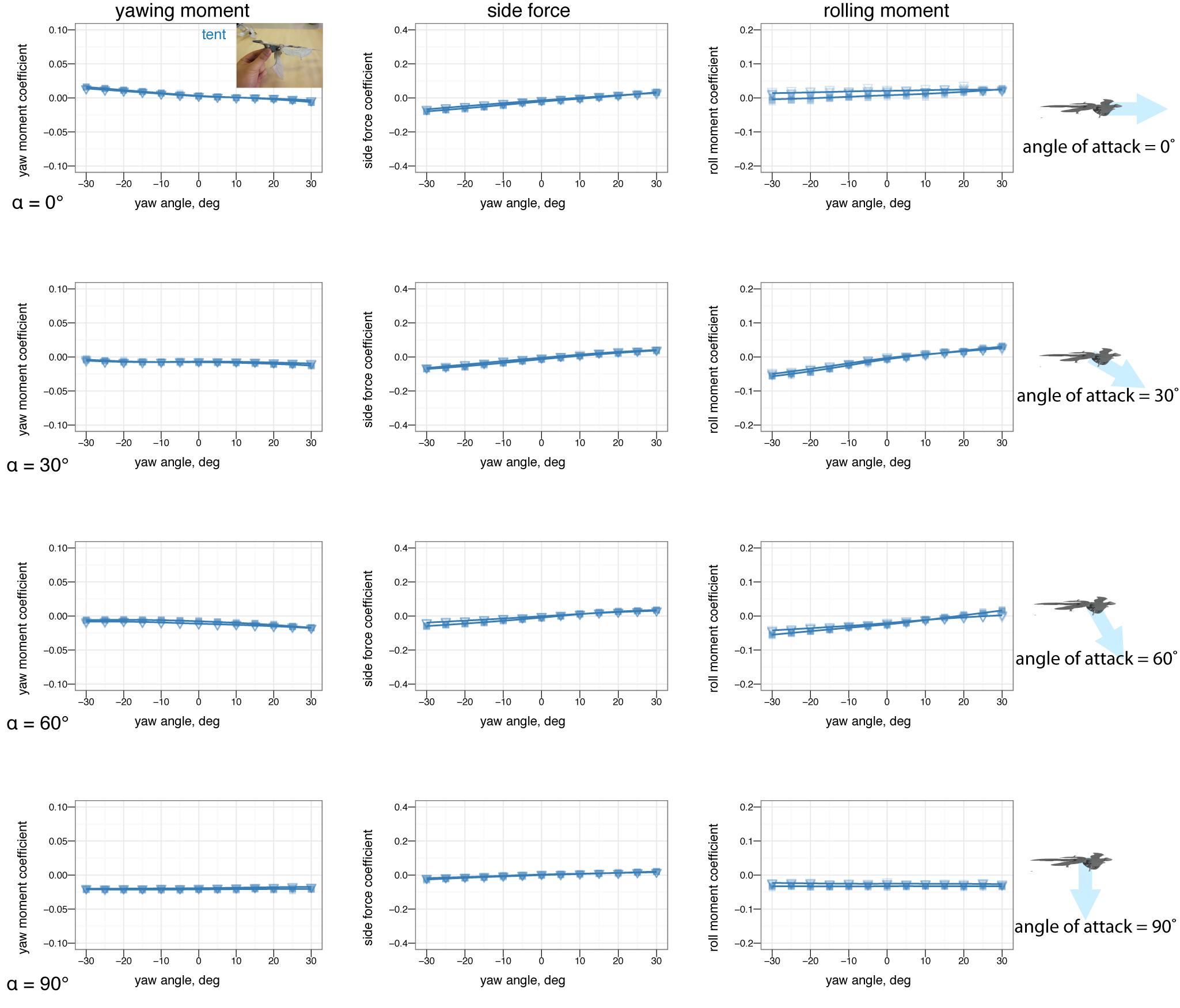

Supplement: Figure S5 — Asymmetric one leg down (leg arabesque ) effect on yaw without leg or tail feathers. Baseline tent position (solid square) versus one leg at 90 mismatch (down arrow). Placing one leg down had little effect; with no leg or tail feathers there is no effect. (TIF) [file pone.0085203.s005.tif]

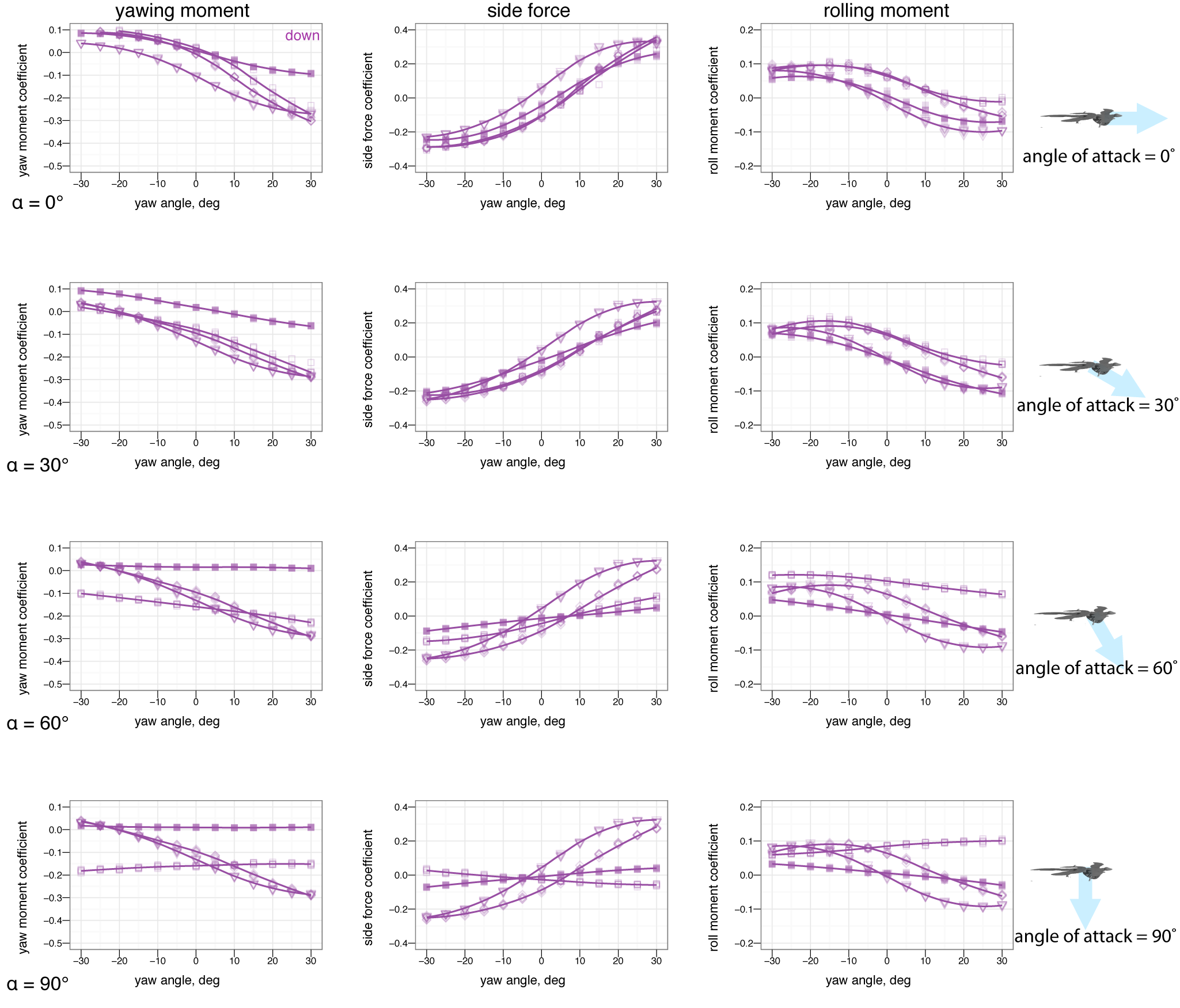

Supplement: Figure S6 — Asymmetric tail movement (lateral bending) effect on yaw, down posture. Baseline down position (solid square), tail 10 left (open square), tail 20 left (open triangle), tail 30 left (open diamond). The tail is effective at creating yawing moments. (TIF) [file pone.0085203.s006.tif]
